# Can Spore Sampler Data Be Used to Predict *Plasmopara viticola* Infection in Vineyards?

**DOI:** 10.3389/fpls.2020.01187

**Published:** 2020-08-13

**Authors:** Chiara Brischetto, Federica Bove, Luca Languasco, Vittorio Rossi

**Affiliations:** Department of Sustainable Crop Production (DI.PRO.VE.S.), Università Cattolica del Sacro Cuore, Piacenza, Italy

**Keywords:** aerobiology, downy mildew, airborne sporangia, secondary infections, spore trap

## Abstract

Grapevine downy mildew (DM) is caused by the dimorphic oomycete *Plasmopara viticola*, which incites epidemics through primary and secondary infection cycles that occur throughout the season. The secondary infection cycles are caused by the sporangia produced on DM lesions. The current research examined the relationship between numbers of airborne sporangia and DM development on grape leaves to determine whether spore sampler data can be useful to predict the potential for secondary infections of *P. viticola*. Three years (2015–2017) of spore sampler data confirmed that sporangia are a common component of the airborne microflora in a DM-infested vineyard and that their numbers depend on weather conditions. For a total of 108 days, leaf samples were collected from the vineyard at 2- to 3-day intervals and incubated under optimal conditions for *P. viticola* infection. The numbers of airborne sporangia sampled on 1 to 7 days before leaf sampling were significantly correlated with the numbers of DM lesions on the leaves. The best correlation (r=0.59), however, was found for the numbers of viable airborne sporangia (SPV), which were assessed by using equations driven by the vapour pressure deficit. In Bayesian and ROC curve analyses, SPV was found to be a good predictor of *P. viticola* infection of grape leaves, with AUROC=0.821 and false positive predictions mainly occurring at low SPV. A binary logistic regression showed that a threshold of 2.52 viable sporangia m^-3^ air day^-1^ enables a prediction of no infection with a posterior probability of 0.870, which was higher than the prior probability of 0.574. Numbers of viable sporangia in the vineyard air is therefore a useful predictor of infection and especially of no infection. The predictor missed some observed infections, but these infections were not severe (they accounted for only 31 of 374 DM lesions).

## Introduction

Downy mildew (DM), which is caused by the oomycete *Plasmopara viticola* (Berk. and Curtis) Berl. and De Toni, is a serious disease of *Vitis vinifera* grapevines. The disease causes both direct and indirect yield losses and develops on leaves, shoots, inflorescences, and clusters. The pathogen has sexual and asexual reproductive stages, which are responsible for primary and secondary infections, respectively (Rossi et al., 2009).

In the traditional view, a DM epidemic begins with the germination of a relatively small number of *P. viticola* oospores, which is followed by a massive clonal multiplication involving secondary infections (Blaeser and Weltzien, 1979; Lafon and Clerjeau, 1988). Molecular studies, however, have documented a continuous input of new genotypes into the epidemic throughout the season (Gobbin et al., 2003; Rumbou and Gessler, 2004; Gobbin et al., 2005), indicating that oospores not only trigger epidemics but also actively contribute to their development (Rossi et al., 2009).

Nevertheless, secondary infection cycles are important in DM epidemics, and their number varies between years or regions depending on weather conditions (Blaeser and Weltzien, 1979; Lafon and Clerjeau, 1988; Schruft and Kassemeyer, 1999). Secondary infections are caused by sporangia produced on DM lesions. Under favourable weather conditions, the pathogen emerges from stomata on the lesion surface and forms sporangia-bearing sporangiophores; this growth and sporulation is visible as a white mold on the abaxial side of the leaf. Sporulation of *P. viticola* is intermittent: it requires darkness and a minimum of 4 h with ≥ 98% relative humidity and ≥ 19°C (Blaeser and Weltzien, 1979). A DM lesion maintains the ability to sporulate for a long time (Kennelly et al., 2007), but sporulation does not occur in sunlight (Gessler et al., 2011); and dry conditions inhibit sporulation but do not necessarily end the ability to sporulate once moist conditions occur (Caffi et al., 2013). The number of sporangia produced on a lesion depends on weather conditions (Arens, 1929; Lalancette et al., 1988; Caffi et al., 2013) and on lesion age (Caffi et al., 2013).

Once mature, sporangia detach from sporangiophores and disperse. The dispersal was commonly considered to be triggered by rainfall (Blaeser and Weltzien, 1978; Kast, 1997), but wind also plays a role (Gilles, 2004); that *P. viticola* sporangia are present in the vineyard air even during periods without rain (Caffi et al., 2013) has been repeatedly demonstrated by aerobiological studies conducted with vineyard spore samplers (Diaz et al., 1997; Diaz et al., 1998; Albelda et al., 2005; Fernandez-Gonzales et al., 2009; Magyar et al., 2009; Fernández-González et al., 2011; Fernández-González et al., 2012; Fernández-González et al., 2019; Martínez-Bracero et al., 2019; Rodríguez et al., 2020). None of these aerobiological studies considered the relationship between the numbers of airborne sporangia in spore samplers and DM development. That relationship, however, has been assessed and used as an action-threshold for disease management in other pathosystems, including hop and lettuce downy mildews, grape powdery mildew, Botrytis leaf blight of onion, and phoma stem canker and light leaf spot of oilseed rape (Royle, 1973; Kremheller and Diercks, 1983; Calderon et al., 2002; Carisse et al., 2005; Falacy et al., 2007; Carisse et al., 2008; West et al., 2008; Carisse et al., 2009a; Carisse et al., 2009b; Thiessen et al., 2016; Thiessen et al., 2017; Dhar et al., 2020). With the advent of molecular tools for the detection and quantification of aerial plant pathogens as well as of methods for automation of spore sampling and wireless reporting of results (West and Kimber, 2015), there is an increasing interest in monitoring airborne inocula and in using this information for disease management decisions (Mahaffee and Stoll, 2016).

The objective of this work was to determine whether the concentration of airborne sporangia of *P. viticola* can be used to predict the potential for secondary infections in vineyards. For this purpose, airborne sporangia were quantified in a vineyard for 3 years, and their numbers were compared with the onset of DM lesions on grape leaves. Numbers of airborne sporangia were considered as both total and viable sporangia, because it is known that sporangia survive on sporangiophores, in air currents, and on plant surfaces for a period that depends on weather conditions, sporangial viability decreasing with increasing temperature and decreasing relative humidity (Blaeser and Weltzien, 1979).

## Materials and Methods

### Experimental Vineyard

The study was conducted for 3 years (2015 to 2017) in an experimental vineyard located on the campus of Università Cattolica del Sacro Cuore, Piacenza (northern Italy, 45° 2’N, 9° 43’E). The experimental plot consisted of two rows of 15 plants each of *V. vinifera* cultivar Barbera, which is susceptible to DM (Rossi and Caffi, 2007)*;* plants were 9 years old in 2015. The vines were separated by 1.1 m in the row and by 1.3 m between rows, and were managed with a single Guyot training system, with no fungicide treatments for the duration of the study. Air temperature (T,°C), relative humidity (RH, %), rainfall (R, mm), leaf wetness (LW, hours), and wind speed (m/s) were recorded by a standard meteorological station (iMetos^®^, Pessl Instruments, Austria) in the experimental vineyard.

### Assessment of Concentration of Sporangia

A volumetric spore sampler (VPPS-2000 Lanzoni, Bologna, Italy) was used to assess concentration of sporangia of *P. viticola*. The spore sampler was placed at 1.5 m above the soil surface between two rows, and was operated from 11 May to 14 September 2015, 11 May to 29 September 2016, and 18 May to 29 September 2017.

**Figure 1:**
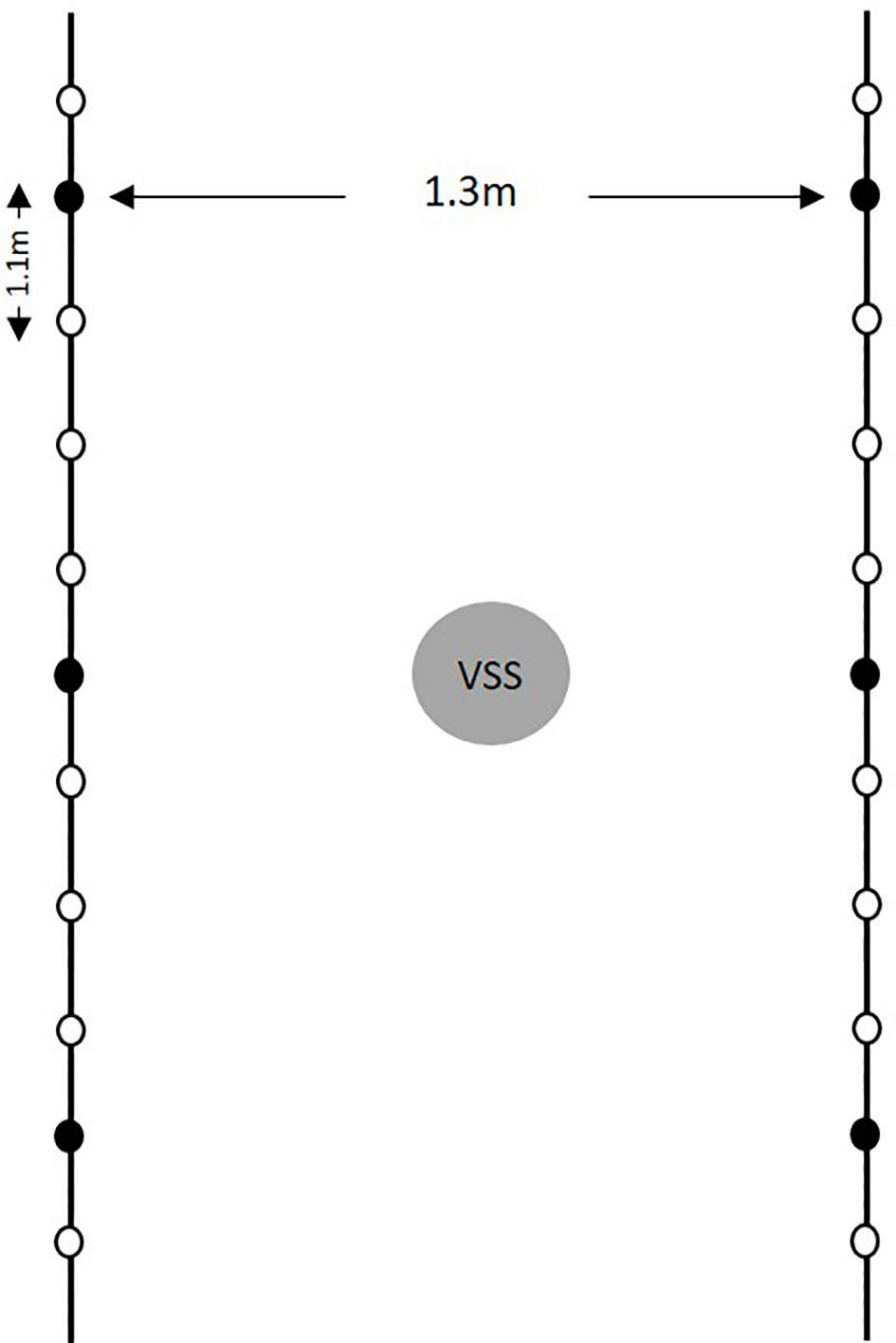
Diagram of the experimental vineyard in the study. Black lines represent the vine rows, and dots indicate plants (the vines is separate by 1.1 m in the row and by 1.3 m between rows); black dots indicate the six plants inoculated with a suspension of *Plasmopara viticola* sporangia; the gray circle represents the volumetric spore sampler (VSS), which was located between the two rows with inoculated vines.

To ensure the presence of *P. viticola* in the vineyard, three vine shoots were inoculated with a suspension of *P. viticola* sporangia. on each of the two rows adjacent to the spore sampler (**Figure 1**). The shoots, which were located at 4 m distance within the rows, were inoculated at 07.00 h (7 am) on 14 May 2015, 19 May 2016, and 18 May 2017 as follows: a hand sprayer was used to spray the abaxial surfaces of the third, fourth, and fifth leaf from the apex of the shoot with the sporangial suspension. The sprayed leaves were immediately covered with polyethylene bags containing a small volume of sterile water and were sealed to maintain a saturated atmosphere; this was done to prevent drying of the inoculum droplets and to favor infection. After 12 h, the bags were removed. The inoculum was prepared by using a needle to collect sporangia from fresh sporulating lesions on leaves of cv. Barbera, which had been grown in pots in a greenhouse, and which had been artificially inoculated with *P. viticola*. The concentration of sporangial suspension was 10^4^ sporangia ml^-1^. This inoculum concentration provided successful inoculation in previous experiments (Caffi et al., 2013).

### Counts of Sporangia

The spore sampler processed air at a flow rate of 10 L min^-1^ and collected sporangia on a Melinex transparent tape (34 cm long and 1.4 cm wide) that was coated with a silicone film. The tape, which was mounted on a cylinder that rotated at 2 mm h^-1^, was replaced every 7 days.

After removal, the tape was brought to the laboratory and cut into seven segments (48 mm per segment), each segment corresponding to 1 sampling day; the i^th^ sampling day began at 10:00 h of the day i^th-1^ and ended at 09:00 h of the i^th^ day. Each segment was then mounted in glycerin jelly (Difco™ gelatin, glycerine 99.5%; liquid phenol 85% sol., and distilled water) on a microscope slide and was protected with a cover glass. Each slide was examined along four longitudinal traverses with a microscope (20× magnification). The *P. viticola* sporangia were enumerated and finally expressed as the total number of sporangia per cubic meter of air on each i^th^ sampling day (SPT_i_, m^-3^ air day^-1^) (**Supplementary Figure 1**).

Seven variables were derived from SPT values: SPT1 and SPT2 to SPT7. SPT1_i_ is the total number of sporangia on day i; SPT2_i_ to SPT7_i_ is the sum of the SPT values on day i and on the days before. SPT7_i_, for example, is the sum of the SPT values on day i and on the six previous days. No longer periods were considered because the infectivity of sporangia strongly decreases when they are more than 7 days old (Kast and Stark-Urnau, 1999).

### Assessment of Viable Sporangia

In each hour of the day, sporangia were assumed to die at a mortality rate (MOR, 0 to 1/24) that depended on the vapour pressure deficit (VPD) and that differed for sporangia still attached to sporangiophores (MOR’) vs. already detached from sporangiophores (MOR”). MOR was calculated by using the equations of Blaeser and Weltzien (1979) as follows:

(1) $$\text{MOR}{’}_{\text{h}}=\frac{1}{24\times (9.27-1.12\times {\text{VPD}}_{\text{h}}+0.04\times {\text{VPD}}_{\text{h}}^{2})}$$

(2) $$\text{MOR}{”}_{\text{h}}=\frac{1}{24\times (5.67-0.47\times {\text{VPD}}_{\text{h}}+0.02\times {\text{VPD}}_{\text{h}}^{2})}$$

where VPD_h_ = T_h_ × (1-RH_h_/100) (Steubing, 1965).

To calculate the daily mortality rate of sporangia (MOR’_i_ and MOR” _i_, 0 to 1) on each i^th^ sampling day, the hourly mortality rate was summed between 10:00 h of the day i^th-1^ and 09:00 h of the i^th^ day. Finally, the daily mortality rate (MOR_i_) was calculated as (MOR’_i_ + MOR”_i_)/2, under the assumption that the airborne sporangia have the same probability of dying before or after their detachment from the sporangiophore.

The number of viable sporangia on each day (SPV_i_, m^-3^ day^-1^) was then calculated by using the following equation:

(3) $${\text{SPV}}_{\text{i}}=\Sigma_{\text{i}=1}^{1-\text{n}}[\text{SP}{\text{T}}_{\text{i}}\times (1-\Sigma_{\text{i}=1}^{1-\text{n}}\text{MO}{\text{R}}_{\text{i}})]$$

where i=1 is the current day, i=-1 defines the day before, i=-2 defines 2 days before, and so on. If $\Sigma_{\text{i}=1}^{1-\text{n}}\text{MO}{\text{R}}_{\text{i}}$ >1, then $\Sigma_{\text{i}=1}^{1-\text{n}}\text{MO}{\text{R}}_{\text{i}}$ = 1.

An example of the use of equation [3] is shown in **Table 1**. On day i=1 (i.e., the current day), SPV_i=1_ = 1.91 m^-3^ air, which is the sum of the sporangia produced on day i=1 (SPT_i=1_ = 1.34), day i=-1 (SPT_i=-1_ = 2.90), and day i=-2 (SPT_i=-2_ = 0.89) that are still viable on the current day; in **Table 1**, these SPV values are 0.85 for day i=1, 0.91 for day i=-1, and 0.15 for day i=-2. The viability of these sporangia results from the application of equation [3] as follows: 0.85 = 1.34×(1-0.363); 0.91 = 2.90×[1-(0.363 + 0.324)]; and 0.15 = 0.89×[1-(0.363 + 0.324+0.149)].

**Table 1:** An example of how the numbers of viable sporangia of *Plasmopara viticola* (SPV) on any day i were determined in the present study.

Day	Airborne sporangia	Weather data			Mortality rate			Viable sporangia (SPV)			
	(SPT)	T(°C)	RH (%)	VPD (hPa)	MOR’	MOR”	MOR	i=-2	i=-1	i=1	Σ
i=-2	0.89	22.4	57	11.6	0.117	0.180	0.149	0.76	–	–	0.76
i=-1	2.90	21.9	64.6	9.59	0.374	0.273	0.324	0.47	1.96	–	2.43
i=1	1.34	23.0	55.77	12.59	0.431	0.295	0.363	0.15	0.91	0.85	1.91

SPV was calculated based on the numbers of total sporangia (SPT) and mortality rates for the sporangia attached to (MOR’) and detached from (MOR”) sporangiophores. Mortality rates were calculated as a function of temperature (T), relative humidity (RH), and vapour pressure deficit (VPD) following equations [1] and [2], respectively. MOR is the average of MOR’ and MOR”.

### Assessment of Downy Mildew Infection

The potential of the airborne sporangia to cause infection was assessed by counting the DM lesions formed on grape leaves that were exposed to sporangia in the vineyard and that were then incubated in the laboratory under optimal conditions for infection. Every 2 or 3 days at 10:00 h, 20 random leaves with no visible DM symptoms were collected in the study area represented in **Figure 1**. Over the 3 years of the study, leaves were collected on 108 dates.

After sampling, leaves were carefully transported to the laboratory, leaf fragments of approximately 8 cm^2^ were excised (20 leaf fragments per sample date, 1 fragment per leaf) with a scissor. Leaf fragments were placed abaxial side up in Petri dishes (four fragments per dish) on wet blotting-paper, and were sprayed with sterile-distilled water (a hand spray bottle was used) so as to form a uniform film of water. Petri dishes were then sealed with Parafilm to maintain a saturated atmosphere and were incubated at 23°C with a 12-h photoperiod. After 24 h, leaf fragment surfaces were dried with sterile filter paper (OIV, 2009), and Petri dishes were sealed and incubated again under the same conditions; during this 24-h period, the viable sporangia present on the leaf surface at the time of their collection in the vineyard were assumed to have caused infection (Unger et al., 2007).

Leaf fragments were observed 10 days after sampling with a stereomicroscope at 10x magnification to detect and enumerate DM lesions (**Supplementary Figure 2**); the number of leaf lesions (NLL) was expressed per fragment, i.e., per 8 cm^2^ of leaf.

### Data Analysis

Pearson’s correlation coefficients were calculated to assess the relationship of SPT1 to SPT7 or SPV with NLL; SPV was selected for further analysis because its correlation with NLL was stronger than those of SPT1 to SPT7 (see Results).

The relationship between the presence of viable sporangia and the development of DM lesions on leaves was analysed by means of a 2×2 contingency table and the chi-square statistic (Quinn and Keough, 2002). For this purpose, SPV and NLL were transformed into dichotomous variables as follows: when SPV>0, S+ (viable sporangia are present in the vineyard at the time of leaf sampling); when SPV=0, S- (viable sporangia are not present); when NLL>0, I+ (infection occurred on leaves sampled from the vineyard and at least one DM lesion developed); NLL=0, I- (no infection). Therefore, the following combinations were possible for each of the 108 leaf-sampling days: (i) viable sporangia were present and infection occurred [S+I+]; (ii) no viable sporangia were present and infection did not occur [S- I-]; (iii) viable sporangia were present and infection did not occur [S+I-]; and (iv) no viable sporangia were present and infection occurred [S-I+].

In a further analysis, numbers of viable sporangia were considered as possible predictors of *P. viticola* infection of the grape leaves sampled from the vineyard in a receiver operating characteristic (ROC) curve (Hanley, 2005). The ROC curve was plotted as the proportion of cases (leaf samples) correctly classified as infected, i.e., as positive (TPP = True Positive Proportion, or sensitivity), as a function of the proportion of cases wrongly classified as non-infected, i.e., as negative (FNP = False Negative Proportion, or 1-specificity), for different cut-off values of SPV. Each point on the ROC curve represents a sensitivity/specificity pair corresponding to a particular cut-off point; the closer the ROC curve is to the upper left corner of the plot, the higher the overall accuracy of the test, i.e., the higher the ratio between the number of cases assigned to the correct category and the number of cases that actually belong to that category (Zweig and Campbell, 1993). The area under the ROC curve (AUROC) and its 95% confidence interval were calculated to measure how well the binary classifier system distinguished between the two groups (infection/no infection). The AUROC ranges between [0.5, 1], and a larger area indicates better performance. The P-value was calculated as the probability that the AUROC is different from the null hypothesis, i.e., that AUROC = 0.5 (the ROC curve coincides with the 1st diagonal) and that the variable under study does not distinguish between the two groups.

Finally, a binary logistic regression (or logit model) was used to predict the odds of having a DM infection (the binary dependent variable, Y) based on the numbers of viable sporangia (the continuous independent variable, X), in the form: P(Y)=1/(1+exp(-(B0+B1×X)). The odds are defined as the probability that a particular SPV results in an infection divided by the probability that it does not result in an infection. Sensitivity, specificity, and accuracy of the model predictions were evaluated with a Bayesian analysis (Yuen and Hughes, 2002). All possible combinations of observed (O) versus predicted (P) infections were organized in a 2x2 contingency table, where the two groups O-P- (no observed and no predicted infection) and O+P+ (yes observed and yes predicted infection) were the correct estimates, while the two groups O-P+ and O+P- were the incorrect ones. To assess the practical advantages of using the model, the posterior probabilities that a particular number of viable sporangia results or does not result in a DM infection were determined as P(O+P+) and P(O-P-), respectively, following Madden et al. (2007), and were compared with the corresponding prior probabilities, P(O+) and P(O-), respectively.

All statistical analyses were carried out using SPSS software (IBM SPSS Statistics, version 25).

## Results

### Weather Data and Numbers of Airborne Sporangia

**Figure 2:**
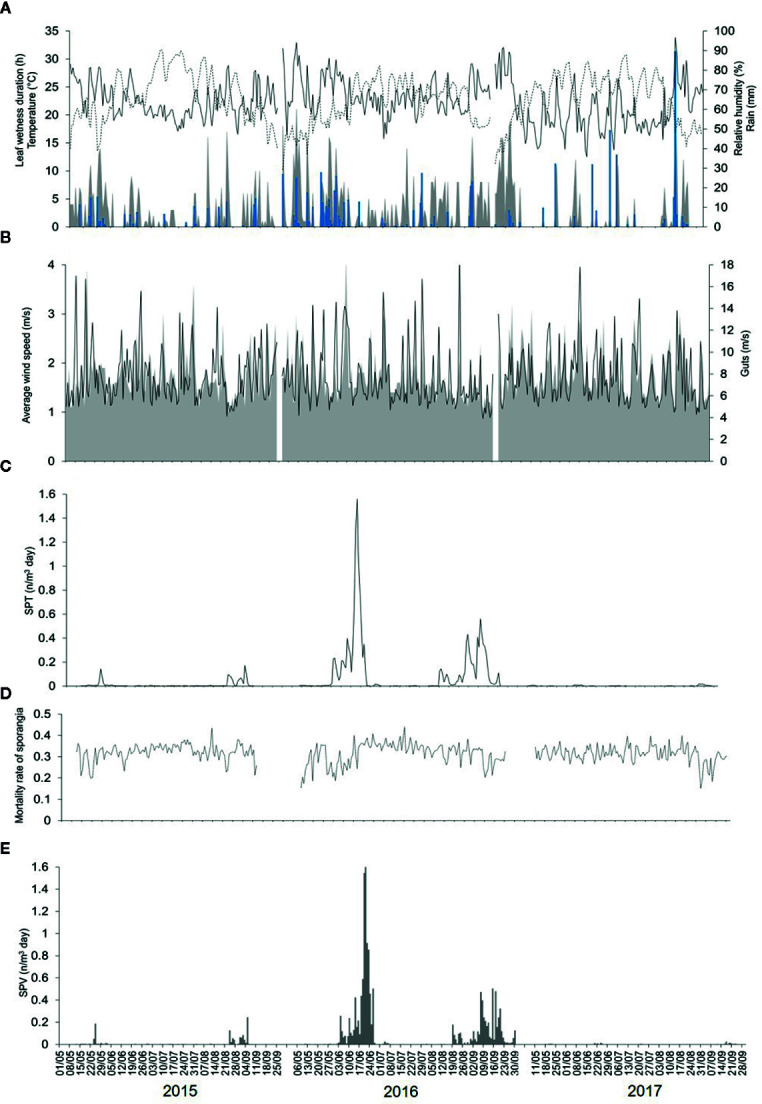
Data for weather (recorded by a weather station in the vineyard) and for numbers of airborne sporangia of *Plasmopara viticola* during the 3 years of the study in the vineyard. **(A)** Temperature (°C, dotted line), relative humidity (%, full line), rainfall (mm, blue bars), and leaf wetness (hours, gray area). **(B)** Average wind speed (m/s, gray area) and gusts (m/s, line). **(C)** Numbers of airborne sporangia of *Plasmopara viticola* m^-3^ day^-1^ (SPT) caught by a volumetric spore sampler in the vineyard (see ****Figure 1****). **(D)** Calculated mortality rate of sporangia. **(E)** Number of viable airborne sporangia m^-3^ day^-1^ (SPV).

Weather data (from 1 May to 30 September, 2015 to 2017) are shown in **Figures 2A, B**; numbers of airborne sporangia are shown in **Figure 2C**.

The 2015 spore sampling season was the hottest and driest of the 3 years. The average temperature was 24.4°C (min = 13.6°C, max = 31.7°C), with an average RH = 63%, a total of 155 mm of rain on 32 rainy days, and a total of 295 h of leaf wetness (**Figure 2A**). The 2016 spore sampling season was the coolest and wettest season; the average temperature was 22.4°C (min = 12.9°C, max = 28.8°C), with an average RH = 68%, a total of 364 mm of rain (more than two times the rain of 2015) on 43 rainy days, and a total of 499 h of leaf wetness (**Figure 2A**). In particular, May and June 2016 were characterised by frequent and intense rain, with prolonged wet periods (**Figure 2A**). The 2017 season was intermediate for temperature, with an average of 23.9°C (min = 14.4°C, max = 30.8°C), and was quite dry; the average RH was 58%, with 314 mm of rain on only 25 rainy days, and a total of only 177 h of leaf wetness (**Figure 2A**). Wind speed and gusts were similar among the 3 years (**Figure 2B**).

Numbers of the airborne sporangia of *P. viticola* reflected the weather conditions, i.e., they were higher in 2016 than in 2015 or 2017 (**Figure 2C**). In 2015, a total of 532 airborne sporangia were detected; airborne sporangia were detected on 116 of the 127 days of the sampling period (91% of the days). On most days in 2015, few airborne sporangia (< 10 sporangia m^-3^ air day^-1^) were detected, but approximately 50 sporangia m^-3^ air were detected on some days, and two peaks occurred on 25 May (189 sporangia m^-3^ air) and 24 August (125 sporangia m^-3^ air). In 2016, a total of 4576 airborne sporangia were detected; airborne sporangia were detected on 122 of the 143 days of the sampling period (85% of the days). There were four peaks in 2016, corresponding to 65, 1547, 244, and 505 sporangia m^-3^ air on 6 June, 20 June, 9 September, and 15 September, respectively. In 2017, a total of 30 airborne sporangia were detected; airborne sporangia were detected on only 72 of 134 days of the sampling period (54% of the days), and there were no peaks.

### Assessment of Viable Sporangia

**Figure 3:**
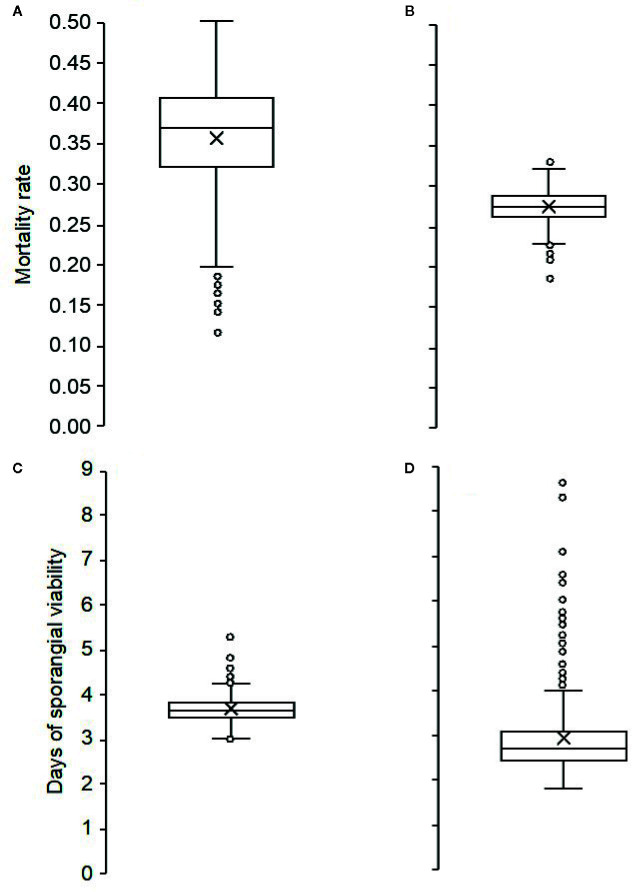
Box plots showing the distribution of: (i) the mortality rates of *Plasmopara viticola* sporangia attached to **(A)** and detached from **(B)** sporangiophores; (ii) numbers of days in which the sporangia attached to sporangiophores **(C)**, or detached from sporangiophores **(D)** remained viable. The horizontal line in the boxes indicates the median; the box includes the 1^st^ and 3^rd^ quartile; dots are outliers.

During the 3 years of the study, the mortality rate of sporangia was highly variable, ranging from 0.15 to 0.44 per day, with an overall average of 0.32 per day. Mortality was higher and more variable (average MOR’=0.36, min=0.12, max=0.55) for sporangia still attached to sporangiophores (**Figure 3A**) than for detached sporangia (average MOR”=0.27, min=0.19, max=0.33) (**Figure 3B**). The dynamics of the mortality rate during the spore sampling periods are shown in **Figure 2D**. Mortality was low in the months of May and June of 2016, which were characterized by cool temperatures and moist conditions (**Figure 2A**). Numbers of viable airborne sporangia during the study are shown in **Figure 2E**.

The duration of sporangia viability ranged from 2 to 9 days for sporangia still attached to sporangiophores (average 3.0 ± 0.04) (**Figure 3C**), and from 3 to 5 days (average 3.7 ± 0.15) for detached sporangia (**Figure 3D**).

### Assessment of DM Infection

**Figure 4:**
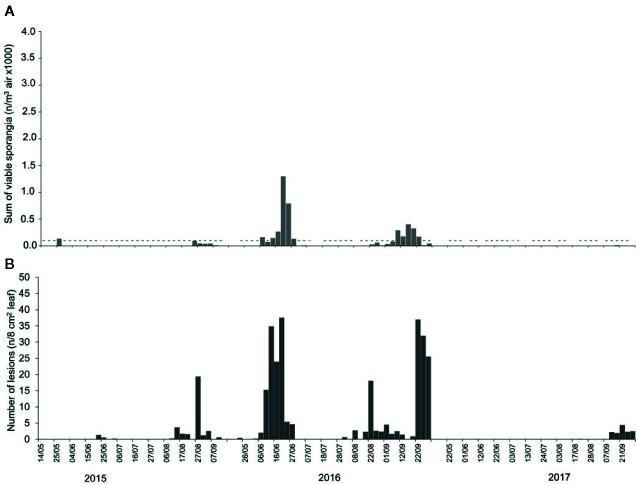
Numbers of **(A)** viable airborne sporangia of *Plasmopara viticola* in the vineyard when grape leaves were sampled to assess downy mildew infection, and numbers of **(B)** downy mildew lesions that developed on the grape leaves sampled from the vineyard. In **(A)**, dashes indicate the days on which viable airborne sporangia were detected, and bars indicate the numbers of viable airborne sporangia/m^-3^ day^-1^.

During 2015, NLL (number of lesions per 8 cm^2^ leaf) averaged 1.54, with the highest value of 22.8 on 27 August. In 2016, NLL averaged 8.20. In May and July of 2016, lesions were absent, and the highest NLL value (46 lesions) occurred on 20 June. However, infections were frequent in August and September of 2016, with peaks of 20 and 37 lesions on 22 August and on 22 September, respectively. During 2017, NLL averaged 0.46. Lesions were almost absent (< 1) from May to August, but a few lesions (< 5) developed in September; the maximum value (5) was on 21 September (**Figure 4B**).

### Relationships Between Numbers of Viable Sporangia and DM Infection

Regardless of the method of calculation, the number of airborne sporangia of *P. viticola* was significantly correlated (P <0.001) with the number of DM lesions on leaves, with correlation coefficients ranging from 0.543 to 0.590 (**Table 2**). The correlation coefficients were lowest for SPT5 to SPT7 and were highest for SPV, i.e., for the number of viable sporangia. Numbers of viable sporangia when grape leaves were exposed in the vineyard and numbers of DM lesions that developed on the leaves are shown in **Figure 4**.

**Table 2:** Person’s correlation coefficients (r) between the number of downy mildew lesions on grape leaves and the number of airborne sporangia of *Plasmopara viticola* when the leaves were exposed in the vineyard.

	SPT1^2^	SPT2	SPT3	SPT4	SPT5	SPT6	SPT7	SPV^3^
r^1^	0.560	0.561	0.561	0.559	0.549	0.543	0.548	0.590

^1^All correlation coefficients were significant at P<0.001; N = 108 for SPT1 to SPT4, and N = 107 for SPT5 to SPT7; ^2^SPT1 to SPT7 are the total numbers of sporangia on the day for SPT1, on the day and the day before for SPT2, etc; SPT7, for example, is the sum of the sporangia on the day and on the six previous days; ^3^SPV is the sum of viable sporangia on a day calculated by using equation [3].

**Figure 5:**
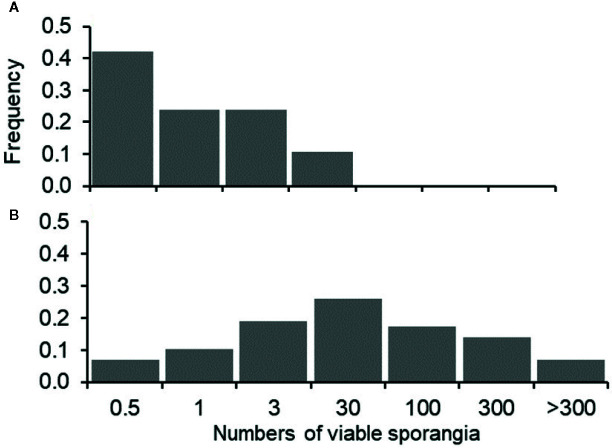
Frequency distribution of the grape leaf samples in which downy mildew lesions did not develop **(A)** or did develop **(B)** in relation to the numbers of viable airborne sporangia of *Plasmopara viticola* (m^-3^ day^-1^, SPV) on leaves that were exposed in the vineyard; numbers of sporangia were grouped into six arbitrary classes: SPV≤0.5; 0.5>SPV≤1; 1>SPV≤3; 3>SPV≤30; 30>SPV≤100; SPV>300.

When the occurrence of infection (estimated as the presence of at least one DM lesion on leaves, i.e., NLL>0: I+; NLL=0: I-)) was predicted based on the presence of viable sporangia (i.e., SPV>0: S+; SPV=0: S-), a relationship was found with Chi-square=3.2 and P=0.074, with [S+I+]=58 (which means that there were 58 periods in which viable sporangia were present and leaves were infected), [S-I-]=8, [S-I+]=4, and [S+I-]=38. Therefore, predictions were wrong on 42 (=4+38) of the 108 sampling dates (39% of the sampling dates), with a prevalence of false positive predictions, i.e., viable sporangia were present that did not result in infection; this was true for 35% of the sampling dates. These false positive predictions, however, mainly occurred when the numbers of viable sporangia were low compared to the numbers of viable sporangia in periods in which grape leaves were infected (i.e., true positive predictions), as shown in **Figure 5**. Therefore, false positive predictions mainly occurred at low SPV.

**Figure 6:**
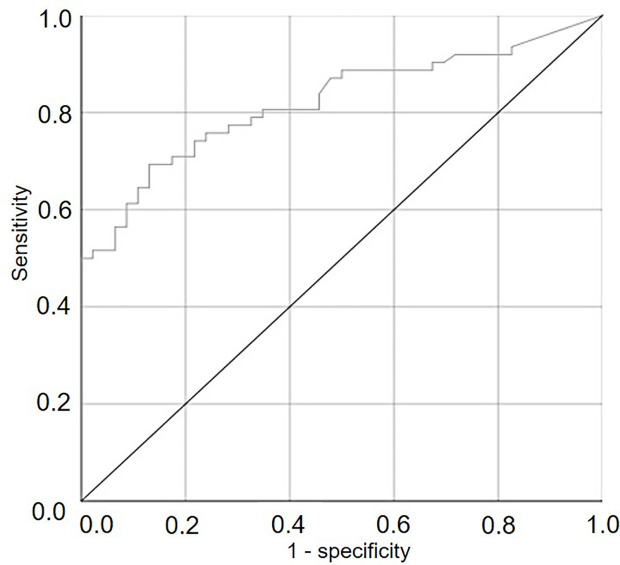
ROC curve showing the trade-offs between sensitivity and specificity for different cut-off values of the numbers of viable airborne sporangia of *Plasmopara viticola* in the vineyard when grape leaves were sampled and used to predict the occurrence of infection on grape leaves. The diagonal is the line of no-discrimination.

The ROC curve (**Figure 6**) generated by using different cut-off values of SPV for predicting the occurrence of infection was significantly different from the line of no-discrimination (the diagonal line) with P<0.001 and AUROC=0.821±0.040, indicating that the number of viable sporangia influenced the binary prediction of infection occurrence.

The binary logistic regression established a relationship between infection occurrence in grape leaves exposed to airborne sporangia of *P. viticola* (as a binary outcome, observed infection yes, O+, or no, O-) and the number of viable sporangia (SPV), i.e., the predictor variable (**Table 3**). When P(Y)=0.5 was used to classify predicted infections, corresponding to SPV=2.52, the logistic regression gave the following results: P+O+=40, P-O-=40, P+O-=6, P-O+=22; therefore, the overall accuracy was 0.74, sensitivity was 0.87, and specificity was 0.65. The likelihood ratio LR+ [= sensitivity/(1 - specificity)] was 2.45, and the likelihood ratio LR- [= (1 - sensitivity)/specificity] was 0.20.

**Table 3:** Coefficients and statistics of the binary logistic regression for predicting *Plasmopara viticola* infection of grape leaves as a function of the numbers of viable airborne sporangia (SPV) when the leaves were exposed in the vineyard.

	Coefficient^1^	S.E.^2^	Wald chi-square^3^	P-value^4^	Exp(B)^5^
SPV	B1 = 0.323	0.109	8.80	0.003	1.381
Constant	B0 = -0.814	0.291	7.81	0.005	0.443

^1^Coefficients of the logistic regression in the form: P(Y)=1/(1+exp(-(B0+B1×X)), expressed in log-odds units, where P(Y) is the probability of infection; ^2^Standard Error of coefficients; ^3^Wald chi-square value and ^4^2-tailed P-value used in testing the null hypothesis that the coefficient is 0; ^5^odds ratios for the coefficients (predictors) as the ratio of the probability of success in predicting infection over the probability of failure.

**Figure 7:**
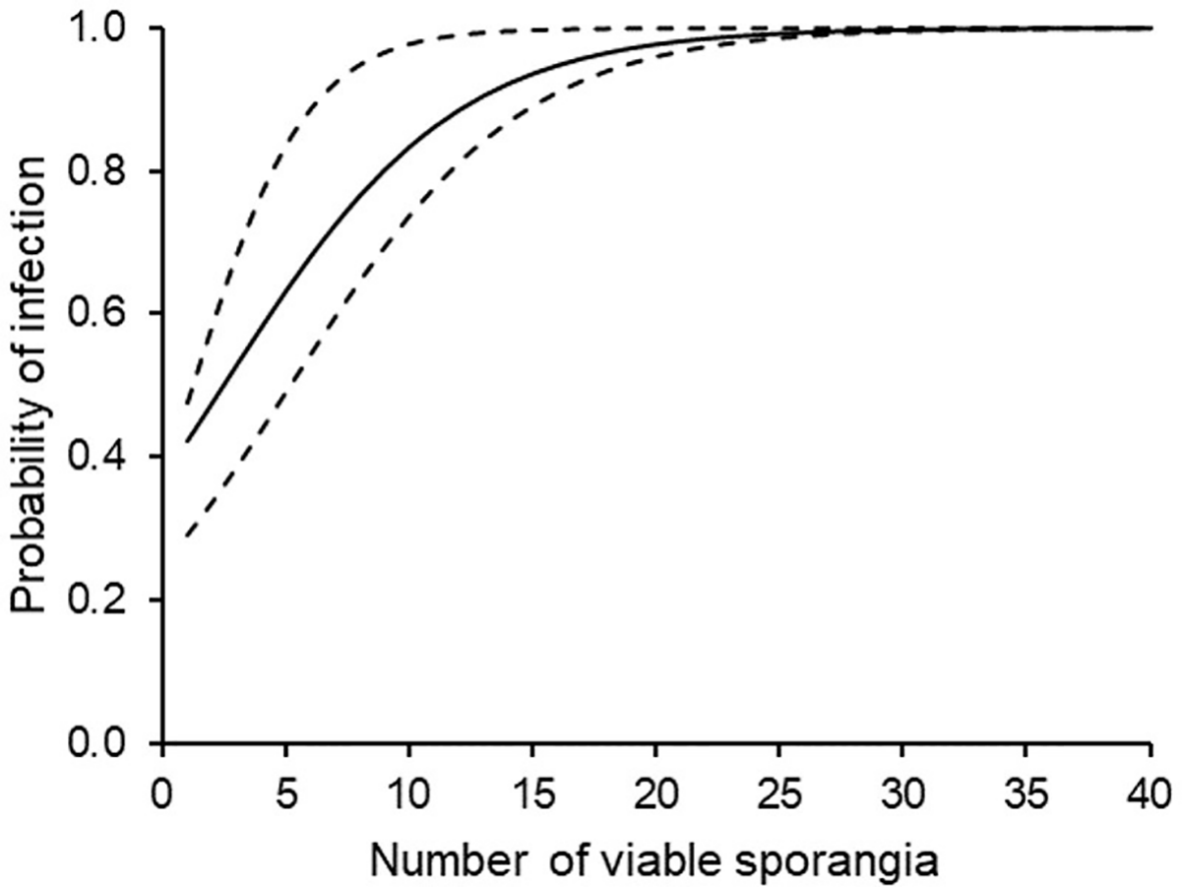
Probability of downy mildew infection on grape leaves that were sampled in the vineyard during periods with different numbers of viable airborne sporangia of *Plasmopara viticola*. The full line is drawn according to the binary logistic regression of **Table 3**; the dotted lines indicate the confidence band calculated by using B1 ± SE and B0 ± SE (see **Table 3**).

This means that, with a threshold of 2.52 (with confidence band 1.21 to 5.16; **Figure 7**) viable sporangia m^3^ air day^-1^ [corresponding to P(Y)=0.5], the probability of predicting a real infection was P(P+O+)=0.645; as a consequence, the probability of predicting an infection that really did not occur was P(P+O-)=0.355. The posterior probability of correctly predicting no infection was P(P-O-)=0.870, and the probability of missing a real infection was P(P-O+)=0.130. Because the prior probabilities were P(O+)= 0.426 and P(O-)= 0.574, the use of viable airborne sporangia increased the ability to correctly predict infection and, even more, to correctly predict no infection.

The classifier based on SPV, therefore, performed better for negative than for positive predictions of *P. viticola* infection. Nevertheless, 22 cases with real infection were missed by the classifier; these missed infections, however, involved only 31 DM lesions, which represented only 8.3% of the total number of lesions observed (374 lesions).

## Discussion

In this work, the dynamics of the secondary inoculum of *P. viticola* were studied during three grape growing seasons (for a total of 410 days) by using a volumetric spore sampler installed between two rows in a vineyard; vines in the rows had been artificially inoculated with *P. viticola* and contained sporulating DM lesions. Sporangia were captured by the sampler in different numbers and on 76.7% of the days, confirming that *P. viticola* sporangia are a common component of the airborne microflora in DM-infested vineyards (Diaz et al., 1997; Diaz et al., 1998; Albelda et al., 2005; Magyar et al., 2009). Our results were especially consistent with those of Fernandez-Gonzales et al. (2009), who reported that *P. viticola* sporangia were detected on 68% of the 176 days on which a volumetric spore sampler was operated. The three growing seasons of the current study, however, differed substantially in weather conditions, and our data confirmed that the abundance of sporangia in the vineyard air strongly depends on weather conditions (Fernandez-Gonzales et al., 2009; Magyar et al., 2009).

Correlations were then determined between the numbers of airborne sporangia detected and the number of DM lesions on grape leaves that were exposed to the inoculum load in the vineyard, transported to the laboratory, and incubated under optimal conditions for the sporangia on the leaf surface to cause infection. The number of lesions on leaves was therefore a proxy for the potential of the sporangia to cause infection. The numbers of sporangia detected in each of seven periods (ranging from 1 to 7 days before leaves were sampled) were all significantly correlated with the numbers of lesions that developed on the leaves. A better correlation was found, however, when only viable sporangia were considered. Although the differences in the correlation coefficients for total and viable sporangia were small (**Table 2**), there are obvious biological advantages that support preference for considering viable instead of total (viable + non-viable) sporangia when the objective is to relate sporangial density to infection occurrence and/or severity (Lalancette et al., 1988; Kast and Stark-Urnau, 1999; Williams et al., 2007; Hong and Scherm, 2020).

Viable sporangia were estimated by calculating a mortality rate (see equations [1] and [2]) based on the equations developed by Blaeser and Weltzien (1979) for sporangia both attached to and detached from sporangiophores and on the studies of Blaeser and Weltzien (1978) of the effects of temperature and relative humidity on spore viability. According to Blaeser and Weltzien (1978), for example, freshly produced sporangia on detached leaves were able to survive less than 24 h at 20°C and 30% RH, and less than 48 h at 20°C and 70% RH. Kast and colleagues found that survival rates calculated based on equations that relied on the epidemiological findings of Blaeser and Weltzien (1978) were consistent with declines in the infectivity of airborne sporangia over time (Kast and Walter, 1994; Kast, 1997; Kast and Stark-Urnau, 1999). The equations of Blaeser and Weltzien (1979), which use vapour pressure deficit (VPD) as the independent variable, enable the calculation of the time sporangia remain viable as a function of temperature and relative humidity. VPD is the difference between the amount of moisture in the air and the amount of moisture that the air can hold when it is saturated at a given temperature (Shuttleworth, 1993). VPD has been used in many scientific fields, including the study of crop physiology (Lawson and Morison, 2004) and plant diseases (Bouma, 2006).

Based on equations [1] and [2], sporangia in the current research were presumed to remain viable for between 2 and 9 days when attached to sporangiophores, and between 3 and 5 days when detached. These results are in substantial agreement with previous findings. Zachos (1959), for example, found that sporangia on sporulating lesions remained viable for 4 to 8 days when protected from direct solar radiation by the canopy and when maximal temperatures did not exceed 22°C; when temperatures ranged from 22 to 25°C, sporangia survived for only 2 days. Kast and Stark-Urnau (1999) found that sporangia that were 1 to 6 days old were able to cause similar infection severity when inoculated onto grape leaves and kept at either 22°C and 26%–34% RH, or at 15–20°C and 60%–80% RH; sporangia rapidly lost viability, however, as they aged beyond 6 days and were unable to infect when they were 10 days old.

Numbers of viable airborne sporangia assessed in this work were found to be good predictors of the occurrence of infection on grape leaves that had been exposed to inoculum in the vineyard. In a ROC curve analysis, the AUROC resulting from plotting sensitivity vs. 1-specificity was 0.821, indicating that the number of viable sporangia enabled the binary prediction of infection occurrence. Sensitivity and specificity used for the calculation of AUROC define the discriminative power of the predictor and indicate the proportion of infected and non-infected leaves correctly identified by the predictor. Sensitivity and specificity, however, explain nothing about the predictive abilities of a positive (infection) or a negative (no infection) outcome.

A binary logistic equation was then calculated. This equation had an overall accuracy of 0.74 in predicting infection or no-infection when the probability threshold for classifying predicted infection was set at 0.5. A threshold of 0.5 corresponded to 2.52 viable sporangia m^-3^ air day^-1^. In general terms, the performance of a predictive algorithm can be evaluated by the sensitivity and specificity, or as likelihood ratios (Yuen and Hughes, 2002). Considering sensitivity (=0.87) and specificity (=0.65), the predictive system based on SPV performed better for negative than for positive prognosis. This was confirmed by the likelihood ratios, which were (LR+)=2.45 and (LR-)=0.20 (Simundic, 2009).

The LR- of this predictive system resulted in a posterior probability of correctly predicting no infection P(P-O-)=0.870, with an increase compared to a prior probability for non-occurrence of infection P(O-)=0.574, which is typical of diseases that are neither infrequent nor frequent for which even predictors with modest performance might be useful (Yuen and Hughes, 2002). This increase in the ability to predict infection can therefore be considered substantial, and the predictive system can therefore be considered useful. As further support of the value of the predictor, in those cases when the binary logistic regression missed real infections, those infections were not severe and accounted for only 8.3% of the total infection observed in our 3-year study.

Based on the results of this work, a threshold of 2.52 viable sporangia m^-3^ air day^-1^ could be used to advise farmers about the risk of DM infection in vineyards with susceptible *V. vinifera* cultivars. To our knowledge, no previous study has defined a threshold of *P. viticola* sporangia numbers that can lead to infection. With respect to other diseases, 50 conidia m^-3^ air day^−1^ was identified as an infection threshold for *Erysiphe necator* (the causal agent of powdery mildew) on grape vines in Quebec (Carisse et al., 2009b), and 15 *Botrytis squamosa* conidia m^-3^ air day^−1^ was identified as an infection threshold for Botrytis blight of onion (Carisse et al., 2005).

The limitations of using data on inoculum presence to support management decisions has recently discussed (West and Kimber, 2015; Mahaffee and Stoll, 2016); these limitations strongly depend on the scale of management that the data are used for. Because the decision-making for in integrated pest management is at the plot level (i.e., at the single vineyard level) (Rossi et al., 2012), this discussion focuses on possible concerns in using the results of the present work for managing DM at the vineyard level. A first concern is the degree to which the spore sampler accounts for the spatial heterogeneity in airborne sporangia, which may be influenced by the spatial heterogeneity of the disease within the vineyard and by the vineyard-to-vineyard movement of the airborne sporangia. Concerning spatial heterogeneity, DM symptoms on leaves were found to be spatially aggregated except when disease incidence was low (Madden et al., 1995), in which case the disease has a random distribution (Seem et al., 1985). The spatial distribution of the secondary inoculum of *P. viticola* within a vineyard, however, is complex and may follow different dispersion patterns that lead to different levels of aggregation (Gobbin et al., 2003). The distance that *P. viticola* sporangia can travel per secondary cycle is usually less than 20 m (Rumbou and Gessler, 2004; Gobbin et al., 2005; Gobbin et al., 2006), but Gobbin et al. (2007), provided evidence of dispersal of up to 130 m. No evidence for long-distance transport has been reported for other oomycetes like *Peronospora tabacina* (Aylor and Taylor, 1982), *Bremia lactucae* (Wu et al., 2001), *Phytophthora infestans* (Aylor et al., 2011), and *Pseudoperonospora cubensis* (Ojiambo and Holmes, 2011).

A second concern is the placement of the sampler(s), which should be based on the biology of the pathosystem being monitored. Location may be critical because the detection of a relatively high concentration of spores can be caused by a small release of spores near the spore trap; similarly, a substantial but local spore release may be missed completely when the spore sampler is far from the inoculum source (West and Kimber, 2015). Spore samplers used to guide powdery mildew management have been located near vines where the disease was the most severe in the previous year (Mahaffee, 2014), because those vines are likely to harbor the overwintering chasmothecia of *Erysiphe necator* that trigger ascosporic infections (Caffi et al., 2008; Rossi et al., 2010; Caffi et al., 2011; Rossi et al., 2011; Thiessen et al., 2016). In the current research, the spore sampler was located between rows of vines showing typical DM symptoms, i.e., in the area of the inoculum source; this location seems also suitable for an extended use of spore samplers for disease management. However, the best number of spore samplers and their locations within the vineyard for the management of grape DM requires further study. For instance, three spore samplers per field have been recommended for the monitoring of *Botrytis squamosa* in onion fields (Carisse et al., 2008), while only one spore sampler per field was recommended for the monitoring of powdery mildew on strawberry (Van der Heyden et al., 2014). The use of mobile samplers mounted on autonomous ground-based mobile vehicles equipped with global positioning systems (GPSs) or mounted on aerial vehicles (West and Kimber, 2015) could also be investigated as an alternative to the use of multiple spore samplers.

A third concern is the time required to analyze spore sampler records, i.e., the records must be analyzed before the farmer must make a decision about crop protection. The time required to analyze spore sampler tapes can be reduced by molecular methods, including quantitative polymerase chain reaction (qPCR) and loop-mediated isothermal amplification (LAMP) (Ward, 2009; Morissette et al., 2012). Both qPCR (Boso et al., 2019) and LAMP (Kong et al., 2016) protocols have been developed for the airborne sporangia of *P. viticola*, but the use of these molecular methods will require research to determine the relationship between the molecular test results and the risk threshold identified in the current study.

Results from this work could be integrated into mathematical models for predicting DM epidemics and for supporting decision making for crop protection, as has been done for other pathosystems (Welham et al., 2004; Carisse et al., 2012; Fall, 2015). Specifically, equations accounting for the mortality of sporangia under variable weather conditions could improve the quantification of the secondary inoculum able to cause infection under suitable weather conditions, and the sporangial threshold could be used to complement risk algorithms for indicating the actual disease risk. Orlandini et al. (2008) introduced into the PLASMO model (Rosa et al., 1993) an equation that was derived from Blaeser and Weltzien (1978) and that accounted for the survival of sporangia; because parameter estimates of this equation were not indicated, however, the equation cannot be used by others. The models VitiMeteo Plasmopara (Bleyer et al., 2008) and RIMpro-Plasmopara (Trapman, 2013) both consider the mortality of sporangia to some extent, but the algorithms were not explicitly described in the reports. Other models such as EPI (Stryzik, 1983), POM (Sung et al., 1990), and Plasmopara Risk Oppenheim (Hill, 1990) do not consider the viability of the secondary inoculum and could therefore benefit from the results of the present work.

## Data Availability Statement

The raw data supporting the conclusions of this article will be made available by the authors, without undue reservation.

## Author Contributions

VR mainly contributed to the conception and the design of the study. CB, LL, and FB carried out the experiments. FB, CB, and VR contributed to the analysis of results. CB, FB, and VR wrote the manuscript. All authors contributed to the article and approved the submitted version.

## Conflict of Interest

The authors declare that the research was conducted in the absence of any commercial or financial relationships that could be construed as a potential conflict of interest.
