# Supplementary material for: Can Spore Sampler Data Be Used to Predict Plasmopara viticola Infection in Vineyards?
Source: Front Plant Sci. 2020 Aug 13;11:1187. doi: 10.3389/fpls.2020.01187 (PMC7438544; doi:10.3389/fpls.2020.01187)
Supplement: Supplementary file 1 [file Image_1.pdf]

Supplementary Material

Supplementary Figures

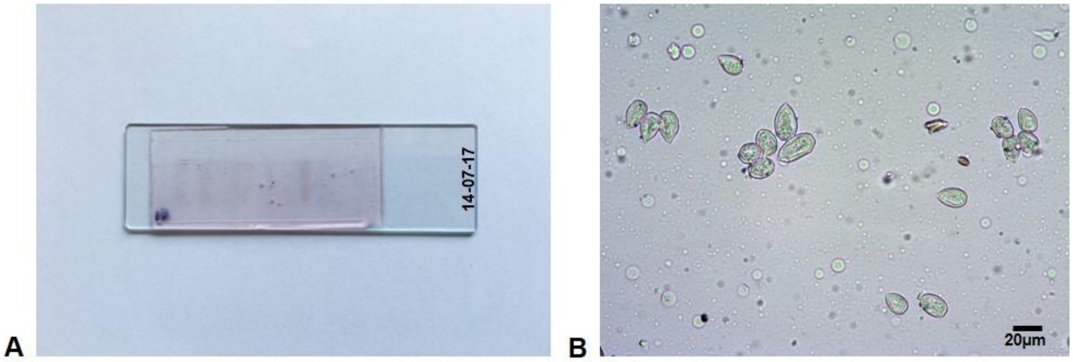

Supplementary Figure 1. A 48 mm segment of Melinex transparent tape coated with silicone film, exposed in vineyard on a spore sampler and mounted in glycerine jelly on a microscope slide (A). *Plasmopara viticola* sporangia (20x magnification) collected from the spore sampler (B).

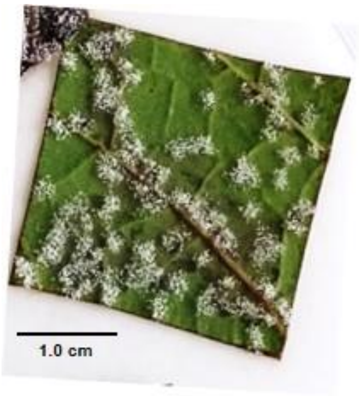

Supplementary Figure 2. Leaf fragment (approximately 8 cm^2) showing downy mildew sporulating lesions after 10 days of incubation at 23°C with a 12-h photoperiod and saturated atmosphere.
